# Genetic Variations in Key MicroRNA Processing Genes and Risk of Head and Neck Cancer: A Case-Control Study in Chinese Population

**DOI:** 10.1371/journal.pone.0047544

**Published:** 2012-10-11

**Authors:** Hongxia Ma, Hua Yuan, Zhiyao Yuan, Chenjie Yu, Ruixia Wang, Yue Jiang, Zhibin Hu, Hongbing Shen, Ning Chen

**Affiliations:** 1 Department of Epidemiology and Biostatistics, MOE Key Laboratory of Modern Toxicology, School of Public Health, Nanjing Medical University, Nanjing, China; 2 Section of Clinical Epidemiology, Jiangsu Key Lab of Cancer Biomarkers, Prevention and Treatment, Cancer Centre, Nanjing Medical University, Nanjing, China; 3 Institute of Stomatology, Nanjing Medical University, Nanjing, China; 4 Department of Otorhinolaryngology, Affiliated Drum Tower Hospital, Medical School of Nanjing University, Nanjing, China; Geisel School of Medicine at Dartmouth, United States of America

## Abstract

MicroRNAs (miRNAs) have been reported to play a key role in oncogenesis. Genetic variations in miRNA processing genes and miRNA binding sites may affect the biogenesis of miRNA and the miRNA-mRNA interactions, hence promoting tumorigenesis. In the present study, we hypothesized that potentially functional polymorphisms in miRNA processing genes may contribute to head and neck cancer (HNC) susceptibility. To test this hypothesis, we genotyped three SNPs at miRNA binding sites of miRNA processing genes (rs1057035 in 3′UTR of *DICER*, rs3803012 in 3′UTR of *RAN* and rs10773771 in 3′UTR of *HIWI*) with a case-control study including 397 HNC cases and 900 controls matched by age and sex in Chinese. Although none of three SNPs was significantly associated with overall risk of HNC, rs1057035 in 3′UTR of *DICER* was associated with a significantly decreased risk of oral cancer (TC/CC vs. TT: adjusted OR  = 0.65, 95% CI  = 0.46–0.92). Furthermore, luciferase activity assay showed that rs1057035 variant C allele led to significantly lower expression levels as compared to the T allele, which may be due to the relatively high inhibition of hsa-miR-574-3p on *DICER* mRNA. These findings indicated that rs1057035 located at 3′UTR of *DICER* may contribute to the risk of oral cancer by affecting the binding of miRNAs to *DICER*. Large-scale and well-designed studies are warranted to validate our findings.

## Introduction

Head and neck cancer (HNC), especially squamous cell carcinoma of the head and neck (SCCHN), is the sixth most common malignancy worldwide, accounting for an estimated 650 000 new cancer cases and 350 000 cancer deaths every year [1], [2]. HNC includes cancers that occur in the paranasal sinuses, nasal cavity, oral cavity, pharynx, and larynx. Tobacco and alcohol consumption have been identified as important caucuses of HNC [1], [2]. Furthermore, studies also reported that genetic factors including family history and genetic variants in multiple biological pathways were involved in the development of HNC [3]. However, the exact mechanism of developing HNC has not been fully explored.

MicroRNAs (miRNAs) are a class of non-coding, single-stranded RNAs of ∼22 nucleotides and act as gene regulators through binding to the 3′-untranslated region (UTR) of protein-coding transcripts, in turn triggering messenger RNA degradation or translational repression [4], [5]. Studies have showed that miRNAs are involved in a variety of biologic processes, including cell cycle regulation, differentiation, development, metabolism and aging. Furthermore, miRNAs have been also linked to the etiology, progression and prognosis of cancer, and miRNA expression profiles can uniquely identify cancer types [6], [7]. The biogenesis of miRNAs is a complex process involving multiple proteins and RNAs [8]. First, large primary precursors of miRNAs (pri-miRNA) are transcribed mainly by RNA polymerase II and cleaved to miRNA precursor (pre-miRNAs) by the nuclear microprocessor complex containing the DROSHA Rnase and the double-strand RNA binding protein DGCR8 [9]. Then, the pre-miRNA is translocated to the cytoplasm through the assistance of Ran-GTPase and Exportin-5 (XPO5), where it is further processed by a protein complex including DICER, leading to the production of double-stranded miRNA duplex.[10]. Subsequently, one strand of miRNA is incorporated into RNA-induced silencing complex (RISC) including HIWI, GEMIN3 and GEMIN4, which mediates expression of target genes. Growing evidence shows that key components in the biosynthetic pathway of miRNA play important roles in the development or prognosis of human cancers including HNC [11], [12], [13].

More recently, it has been reported that the presence of single nucleotide polymorphisms (SNPs) in microRNA genes, their processing machinery and target binding sites affects the susceptibility to cancers by modulating expression levels of miRNAs and miRNA–mRNA interactions [14]. Among those, some studies have focused on the effect of SNPs in miRNA-binding sites (miRNA-binding SNPs) of miRNA processing genes and cancer risk. For instance, one study with 130 oral premalignant lesions (OPLs) cases and 136 controls found that the variant allele of rs3742330 in 3′UTR of *DICER* significantly increased the risk of OPLs in Americans [15]. Two other studies reported that rs11077 in 3′UTR of *XPO5* and rs14035 in 3′UTR of *RAN* were associated with risk of esophageal cancer [16] or renal cell carcinoma [17]. However, to date, there is no report about the relation between genetic variations in miRNA biogenesis genes and HNC risk. In this study, we hypothesized that potentially functional SNPs in 3′UTR of miRNA biogenesis genes might modify HNC risk in Chinese population. To test this hypothesis, we performed a genotyping analysis of three SNPs (rs1057035 in *DICER*, rs3803012 in *RAN* and rs10773771 in *HIWI*) with a case-control study of 397 HNC cancer cases and 900 healthy controls in Han Chinese and evaluated their individual and joint effect on HNC risk. To clarify the functional relevance of the selected SNPs with HNC cancer, we also examined the luciferase activity of *DICER* rs1057035 (T and C alleles) by the cloning and reporter gene assay, assessing the effects of the SNP on the binding of special miRNAs to the 3′UTR of *DICER*.

**Table 1:** Distributions of selected variables in HNC cases and cancer-free controls.

Variables	Cases	Controls	*P* a
	N (%)	N (%)	
All subjects	397(100)	900(100)	
Age, yr			0.637
≤60(median)	209(52.6)	461(51.2)	
>60(median)	188(47.4)	439(48.8)	
Sex			0.263
Females	136(34.3)	280(31.1)	
Males	261(65.7)	620(68.9)	
Smoking status b			0.580
No	212(53.7)	498(55.3)	
Yes	183(46.3)	402(44.7)	
Drinking status b			<0.001
No	217(54.9)	609(67.7)	
Yes	178(45.1)	291(32.3)	
Tumor sites			
Oral cavity	293(73.8)		
Oropharynx	6(1.5)		
Larynx	88(22.2)		
Other^c^	10(2.5)		
Histology			
Squamous	335(84.4)		
Other d	62(15.6)		

a Two-sided *χ^2^* test.

b 2 subjects were absent of smoking information and 2 subjects were absent of drinking information.

c Including nasal sinuses, parotid and salivary gland.

d Including adenocarcinoma, undifferentiated carcinoma and undetermined cancer.

## Materials and Methods

### Ethics Statement

This study was approved by the institutional review board of Nanjing Medical University. The design and performance of current study involving human subjects were clearly described in a research protocol. All participants were voluntary and would complete the informed consent in written before taking part in this research.

**Table 2:** Variant genotypes of miRNA processing genes and their associations with risk of HNC.

SNPs	Genotype	Controls	Overall Cases	Adjusted OR	*P* a	Oral cavity cases	Adjusted OR	*P* a	Other cases	Adjusted OR	*P* a
		N(%)	N(%)	(95% CI) a		N(%)	(95% CI) a		N(%)	(95% CI) a	
**rs1057035**											
	TT	678 (76.1)	317 (80.5)	1.00		240 (82.8)	1.00		77 (74.0)	1.00	
	TC	204 (22.9)	75 (19.0)	0.78 (0.58–1.06)	0.108	48 (16.6)	0.65 (0.46–0.93)	**0.019**	27 (26.0)	1.18 (0.73–1.90)	0.498
	CC	9(1.0)	2(0.5)	0.44 (0.09–2.10)	0.306	2(0.7)	0.61 (0.13–2.88)	0.529	-	-	-
	TC/CC vs. TT	213 (23.9)	77 (19.5)	0.77 (0.57–1.03)	0.080	50 (17.3)	0.65 (0.46–0.92)	**0.016**	27 (26.0)	1.13 (0.70–1.82)	0.615
	Additive			0.77 (0.58–1.02)	0.064		0.67 (0.48–0.93)	**0.018**		1.06 (0.68–1.67)	0.794
**rs3803012**											
	AA	799 (89.6)	344 (88.0)	1.00		253 (88.2)	1.00		91 (87.5)	1.00	
	AG	91 (10.2)	45 (11.5)	1.15 (0.78–1.68)	0.481	32 (11.2)	1.11 (0.72–1.72)	0.631	13 (12.5)	1.37 (0.73–2.58)	0.329
	GG	2(0.2)	2(0.5)	2.62 (0.36–19.30)	0.343	2(0.7)	3.76 (0.50–28.5)	0.200	-	-	-
	AG/GG vs. AA	93 (10.4)	47(12)	1.18 (0.81–1.72)	0.397	34 (11.9)	1.16 (0.76–1.78)	0.489	13 (12.5)	1.34 (0.71–2.51)	0.367
	Additive			1.20 (0.83–1.71)	0.330		1.20 (0.80–1.80)	0.370		1.29 (0.70–2.37)	0.217
**rs10773771**											
	TT	310 (34.8)	143 (36.5)	1.00		111 (38.4)	1.00		32 (31.1)	1.00	
	TC	399 (44.7)	183 (46.7)	0.99 (0.76–1.29)	0.936	129 (44.6)	0.91 (0.67–1.23)	0.528	54 (52.4)	1.21 (0.76–1.94)	0.421
	CC	183 (20.5)	66 (16.8)	0.78 (0.55–1.11)	0.169	49 (17.0)	0.76 (0.51–1.12)	0.160	17 (16.5)	0.82 (0.44–1.52)	0.526
	TC/CC vs. TT	582 (65.2)	249 (63.5)	0.92 (0.72–1.19)	0.539	178 (61.6)	0.86 (0.65–1.14)	0.292	71 (68.9)	1.09 (0.70–1.70)	0.715
	Additive			0.90 (0.76–1.07)	0.222		0.88 (0.73–1.06)	0.167		0.94 (0.71–1.26)	0.689

a Adjusted for age, sex, smoking status and alcohol status.

### Study population

All newly and histologically confirmed HNC patients were consecutively recruited from Jiangsu Stomatological Hospital and the First Affiliated Hospital of Nanjing Medical University, Nanjing, China, since January 2009 to April 2011. Exclusion criteria included second HNC primary tumors or metastasized cancer from other organs. 420 cases were initially contacted for participation and approximately 95% of eligible cases (397 cases) agreed to participate in the study. Cancer-free controls frequency-matched to the cases on age (±5 years) and sex were randomly selected from a cohort of more than 30,000 participants in a community-based screening program for non-infectious diseases in the Jiangsu Province, China. All participants were genetically unrelated, ethnic Han Chinese population. When written informed consent was obtained, a structured questionnaire was used by trained interviewers to collect information on demographic data and environmental exposure history, such as age, sex, smoking and drinking consumption. After interview, approximately 5-ml venous blood sample was collected from each participant. Finally, a total of 397 cases and 900 controls completed the interview and donated the blood sample.

**Figure 1:**
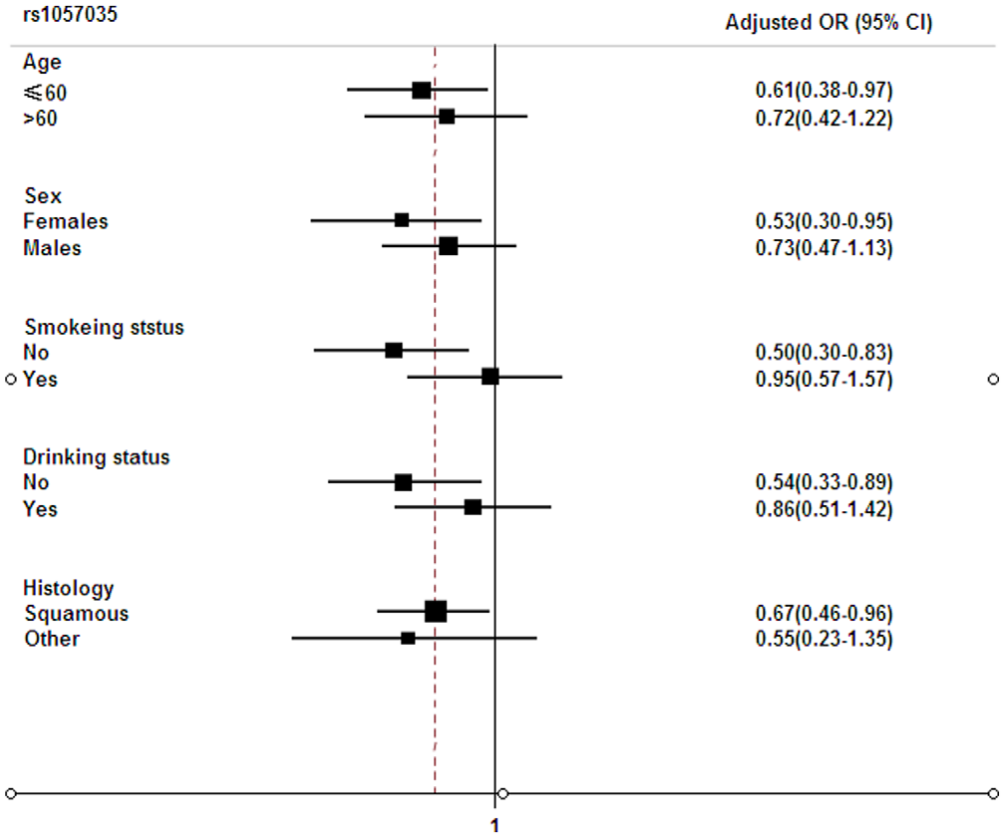
Stratified analyses on associations between variant genotypes of rs1057035 and oral cancer risk (Dominant model).

### SNP selection and genotyping

Eight key miRNA biogenesis genes (*DROSHA*, *DGCR8*, *XPO5*, *DICER*, *HIWI*, *GEMIN3*, *GEMIN4* and *RAN*) were selected through an extensive literature search. For each gene, we first used the NCBI dbSNP database (http://www.ncbi.nlm.nih.gov/ build 131) to search SNPs that localized at 3′ UTR of genes and found 17 SNPs with minor allele frequency (MAF)>0.05 in Chinese population. Then, two web-based analysis tools were used to predict the effect of 17 SNPs on miRNA binding (http://snpinfo.niehs.nih.gov/snpfunc.htm; http://miracle.igib.res.in/dbSMR) [18], [19] and only 4 SNPs with consistent findings in both tools were remained. Among these SNPs(rs11077, rs1057035, rs10773771, and rs3803012), rs11077 was excluded because the further search by mirBase database (http://www.mirbase.org/search.shtml) showed that this SNP was not located in the binding region for any miRNA. As a result, we identified 3 miRNA-binding SNPs in three genes (rs1057035 in *DICER*, rs3803012 in *RAN* and rs10773771 in *HIWI*). Among those, rs1057035 was predicted to influence has-miR-574-3p binding, rs3803012 influencing has-miR-199a-3p binding and 10773771 influencing has-miR-1264 binding.

**Figure 2:**
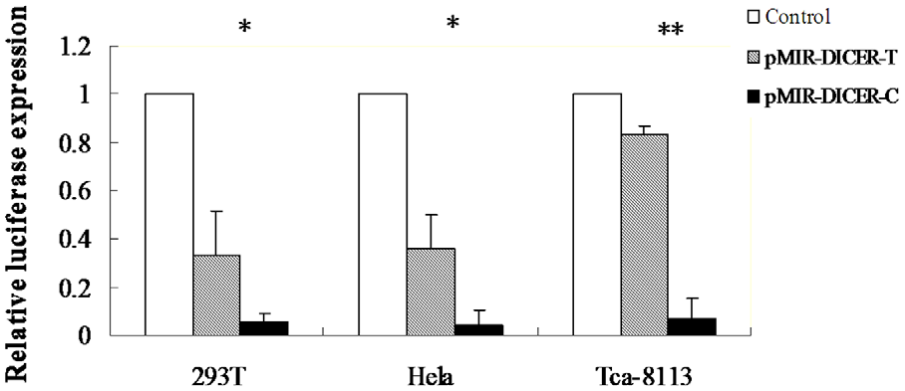
DICER 3′UTR luciferase plasmids (T allele or C allele) and chemically synthesized mature has-miR-574-3p expression plasmids in 293T, Hela and Tca8113 cells. (*, P<0.05; **, P<0.001).

Genomic DNA was isolated from leucocytes of venous blood by proteinase K digestion and phenol/chloroform extraction. The genotyping of SNPs was performed by using TaqMan allelic discrimination Assay on an ABI 7900 system (Applied Biosystems, Foster city, CA). Genotyping was performed without knowing the subjects′ case or control status, and two negative controls in each 384-well format were used for quality control. The genotyping results were determined by using SDS 2.3 Allelic Discrimination Software (Applied Biosystems). The concordance achieved 100% for the duplicates of 5% of samples for each SNP.

### Luciferase reporter assay to determine effect of miRNA binding on DICER expression

The 3′UTR region of *DICER* containing the putative recognition site rs1057035 was amplified from the sample DNA, then cloned into the pMIR-REPORT^TM^ (Applied Biosystems) vector with Mlu I and Hind III digestions. The primers were: GTAGACGCGTTCAACAGCAAACTTCAGCC (sense) and TCCAAAGCTTGGCAGGGTATCAGAATCTTT (antisense). Plasmids containing the different alleles of rs1057035 were generated using site-specific mutagenesis. All constructs used in this study were restriction mapped and sequenced to confirm their authenticity.

Tca-8113 (a human oral squamous cell carcinoma cell line)[20], 293T (a human embryonic kidney cell line) and Hela (a human cervical carcinoma cell line) cells were cultured in Dulbecco's modified Eagle's medium with high glucose (Gibco) supplemented with 10% heat-inactivated fetal bovine serum (Gibco) and 50 ug/ml streptomycin (Gibco) at a 37°C incubator supplemented with 5% CO2. Cells were seeded at 1×10^5^ cells per well in 24-well plates (BD Biosciences, Bedford, MA). Twenty-four hours after the plating, cells were transfected by Lipofectamine 2000 according to manufacturer's suggestion (Invitrogen). The *DICER* 3′UTR luciferase plasmids (different alleles) and chemically synthesized mature hsa-miR-574-3p were cotransfected respectively. The pRL-SV40 plasmid (Promega) was cotransfected as a normalizing control. Six replicates for each group and the experiment repeated at least three times. After 24 hours of incubation, cells were collected and analyzed for luciferase activity with the Dual-Luciferase Reporter Assay System (Promega).

### Statistical analysis

The Hardy-Weinberg equilibrium was tested by a goodness-of-fit χ^2^ test to compare the observed genotype frequencies with the expected ones among the control subjects. Distributions of selected demographic variables, risk factors, and frequencies of variant genotypes between the cases and the controls were evaluated by using the χ^2^ test. The associations of variant genotypes with HNC risk were estimated by computing odds ratios (ORs) and 95% confidence intervals (CIs) from both univariate and multivariate logistic regression analyses. The heterogeneity between subgroups was assessed with the Chi-square-based Q test and the heterogeneity was considered significant when *P*<0.05. The possible gene-gene and gene-environment (i.e. smoking and drinking status) interactions were evaluated by the logistic regression models. All the statistical analyses were performed with Statistical Analysis System software (v.9.1 SAS Institute, Cary, NC). Two-sided tests were generally used for statistical analysis and *P*<0.05 was considered as the level of statistical significance.

## Results

The selected characteristics of the cases and the controls are shown in **Table** **1**. There were no significant differences in the distributions of age, sex and smoking between the cases and the controls (*P* = 0.637, 0.263 and 0.580, respectively), and the mean age was similar (59.98±11.94 years for the cases and 60.65±7.99 years for the controls). As expected, more drinkers were observed in the case group compared with that in the control group (45.1% vs. 32.2%, *P*<0.001). Of the 397 cases, 293(73.8%) were with the tumor of oral cavity, 6(1.5%) with oropharynx, 86(22.2%) with larynx and 10(2.5%) with others. Furthermore, 335 cases (84.4%) presented with squamous cell carcinoma.

SNPs were at Hardy-Weinberg equilibrium (*P* = 0.14 for rs1057035 and *P* = 0.73 for rs3803012, respectively) except rs10773771 (*P* = 0.01). The genotype and allele distributions of *DICER* rs1057035, *HIWI* rs10773771 and *RAN* rs3803012 in cases and controls are shown in Table 2. The single locus analyses revealed that genotype distributions of these three polymorphisms were not significantly different between overall cases and controls (*P* = 0.080 for rs1057035, *P* = 0.397 for rs3803012 and *P* = 0.539 for rs10773771, respectively). To further investigate the modifying effects of three variants on risk of HNC with different tumor sites, we conducted the stratification analysis by oral cavity and non-oral cavity cancers. As shown in Table 2, rs1057035 variant genotypes were associated with a significantly decreased risk of oral cancer (TC vs. TT: adjusted OR  = 0.65, 95% CI  = 0.46–0.93, adjusted *P* = 0.019; TC/CC vs. TT: adjusted OR  = 0.65, 95% CI  = 0.46–0.92, adjusted *P* = 0.016; additive model: adjusted OR  = 0.67, 95% CI  = 0.48–0.93; adjusted *P* = 0.018). None of significant associations was observed between the genotypes of two other SNPs (rs3803012 A>G, rs10773771 G>A) and risk of HNC with different sites (**Table** **2**).

We further conducted the stratification analysis on the associations between rs1057035 and oral cancer risk by age, sex, smoking, drinking and histology. As shown in **Figure** **1**, the decreased risk of oral cancer associated with the variant TC/CC genotypes was significant among the younger (adjusted OR  = 0.61; 95% CI  = 0.38–0.97), females (adjusted OR  = 0.53; 95% CI  = 0.30–0.95), non-smokers (adjusted OR  = 0.50; 95% CI  = 0.30–0.83) and non-drinkers (adjusted OR  = 0.54; 95% CI  = 0.33–0.89), compared with the TT genotype. However, heterogeneity test showed that there was no significant heterogeneity (*P*>0.05) in every two stratums. We also evaluated the effect of the addition of alcohol, smoking and gender on ORs and found that these were no differences if these variables were included in the models or not included (**Table S1**). Additionally, no significant results were observed for gene-gene or gene-environment (i.e. smoking status and alcohol status) interactions (data not shown).

Because rs1057035 showed significant association with oral cancer risk and was predicted to locate at the putative miRNA binding site (hsa-miR-574-3p), it is interesting to test whether rs1057035 influences the targeting of hsa-miR-574-3p to *DICER* mRNA in vitro. Thus, we generated reporter genes for *DICER* 3′UTR (C or T allele) that were cotransfected with chemically synthesized mature hsa-miR-574-3p in Tca8113 oral squamous cell carcinoma cell lines. As shown in **Figure** **2**, cotransfection of hsa-miR-574-3p significantly inhibited the expression of the reporter carrying rs1057035 C allele but not the T allele (293T cell line: 0.057±0.002 for C allele versus 0.333±0.089 for the T allele, *P* = 0.032; Hela cell line: 0.041±0.007 for C allele versus 0.357±0.257 for the T allele, *P* = 0.030; Tca8113 cell line: 0.072±0.067 for C allele versus 0.833±0.120 for the T allele, *P*<0.001). The results suggested that the rs1057035 variant allele could differentially affect the targeting of hsa-miR-574-3p to 3′UTR of *DICER* in oral cancer cells.

## Discussion

In this case-control study of 397 HNC patients and 900 cancer-free controls in a Chinese population, we investigated the associations between three SNPs in 3′UTR of miRNA biosynthesis genes and risk of HNC. Although we did not find evidence for a main effect of each SNP on overall HNC risk, the subgroup analysis of HNC showed that variant genotypes of rs1057035 were associated with the risk of HNC arising at oral cavity. Additional functional analyses showed that rs1057035 C allele led to significantly lower luciferase activity, compared with the T allele. These findings suggested, for the first time, that potentially functional polymorphisms of *DICER* may play a role in the development of HNC, particularly of those tumors arising at oral cavity.

DICER is a enzyme responsible for the cleavage of miRNA precursors and has previously been implicated in the oncogenic process of several cancers [21], [22], [23]. Evidence indicates that *DICER* may have different roles in the tumorigenesis of different cancers. For example, some studies showed that lower levels of *DICER* mRNA expression were associated with the development of lung cancer [24] and the prognosis of ovarian cancer [11]. However, studies also demonstrated that elevated expression levels of *DICER* were correlated with increased cell proliferation of oral cancer cells [12]. In our study, we found that rs1057035 variant C allele led to significantly lower expression levels of *DICER* and exhibited a protective effect on oral cancer susceptibility, which were consistent with the findings in oral cancer cells [12]. Furthermore, we found that the protective effect of rs1057035 variant genotypes was statistically significant in some subgroups, such as females, non-smokers and non-drinkers. However, heterogeneity test showed no significant heterogeneity (*P*>0.05) between every two stratums, implying that there was no risk effect modification by these variables under investigation.

The significant SNP (rs1057035) identified in our study is located in the 3′ UTR of *DICER* and predicted to affect the binding of hsa-miR-574-3p. Hsa-miR-574-3p is evolutionarily conserved at the nucleotide level from flies to humans. Although the function of hsa-miR-574-3p is not clear, it is predicted to target several hundreds of human genes [25]. Our results indicated that the *DICER* rs1057035 C allele might affect the targeting of hsa-miR-574-3p and result in the decreased expression of *DICER* mRNA in oral cancer cells, which may be a possible underlying mechanism for the observed association between rs1057035 and the decreased risk of oral cancer. However, we did not find any association between rs1057035 and overall risk of HNC cancer or non-oral cancer risk in our study. The pathogenesis of oral cancer may be slightly different from other cancers arising at head and neck. For example, recent epidemiologic and molecular studies have identified high-risk types of human papillomavirus (HPV) as the potential etiologic factor for HNC, but it is more prominent in the oropharynx cancer than that in the oral cavity cancer [3], [26].

Additionally, we also found no significant associations between SNPs in 3′UTR of *RAN* and *HIWI* and HNC risk. RAN is a unique member of the Ras superfamily of GTPases, which is essential to the transportation of pre-miRNAs from nucleus to cytoplasm through the nuclear pore complex in a GTP-dependent manner [27]. HIWI is a part of the RNA Induced Silencing Complex (RISC) and a key regulator of stem cell pluripotency by controlling cell self-renewal and differentiation [28]. Some studies have investigated the associations between polymorphisms of these two genes and risk of several cancers, such as esophageal cancer, bladder cancer and renal cell carcinoma, but the results were inconsistent [15], [16], [17], [29]. In our study, we first found that *RAN* rs3803012 and *HIWI* rs10773771 variant genotypes were not associated with risk of HNC, suggesting these SNPs might not modulate the susceptibility of HNC in Chinese population.

Several potential limitations of the present study warrant considerations. First of all, a relatively small sample size may limit the statistical power of our study, especially in stratification analysis by tumor sites. We evaluated the statistical power for the effect of rs1057035 by the PS software (Power and the Sample Size Calculations version 1.0.17) and found that in the case of OR = 0.65 (dominant model), our sample could reach 78.8% of the statistical efficacy. Secondly, it is a hospital-based case-control study and inherent selection bias cannot be completely excluded. However, we applied a rigorous epidemiological design in selecting study subjects and used further statistical adjustment for known risk factors to minimize potential biases. Thirdly, since the intensity and duration of smoking and drinking were absent in this study, it was difficult to do future analysis for such exposure variables. Last, we only selected miRNA-binding SNPs for genotyping and could not evaluate the relationship between other potentially functional SNPs in miRNA processing genes and HNC risk. Thus, larger, well-designed epidemiological studies with ethnically diverse populations are warranted to confirm and expand our findings.

## Supporting Information

Table S1 **The analysis for the effect of variables on ORs in models.** NOTE:^ a^ Likelihood ratio test was used to check the difference between −2*log Likelihood of the two models. ^b^ Compared to the first model including age, gender, smoking and drinking.
(DOCX)
