# Supplementary material for: Genetic Variations in Key MicroRNA Processing Genes and Risk of Head and Neck Cancer: A Case-Control Study in Chinese Population
Source: PLoS One. 2012 Oct 11;7(10):e47544. doi: 10.1371/journal.pone.0047544 (PMC3469541; doi:10.1371/journal.pone.0047544)
Supplement: Table S1 — The analysis for the effect of variables on ORs in models. NOTE: a Likelihood ratio test was used to check the difference between −2*log Likelihood of the two models. b Compared to the first model including age, gender, smoking and drinking. (DOCX) [file pone.0047544.s001.docx]

# Supplemental Table S1. The analysis for the effect of variables on ORs in models

| **SNPs** | **Adjusted variables in models** | **Adjusted OR (95%CI)** | **Adjusted *P*** | ***P* for comparisons of different models ^a^** |
| --- | --- | --- | --- | --- |
| **rs1057035**  **TC/CC vs. TT** | age, gender, smoking and drinking | 0.65(0.46-0.92) | 0.016 |  |
|  | age, gender and smoking | 0.64(0.46-0.91) | 0.014 | 0.429 ^b^ |
|  | age, gender and drinking | 0.65(0.46-0.93) | 0.015 | 0.576 ^b^ |
|  | age, smoking and drinking | 0.66(0.47-0.94) | 0.019 | 0.278 ^b^ |
| **rs3803012**  **AG/GG vs. AA** | age, gender, smoking and drinking | 1.16(0.76-1.78) | 0.489 |  |
|  | age, gender and smoking | 1.14(0.75-1.73) | 0.551 | 0.254 ^b^ |
|  | age, gender and drinking | 1.15(0.75-1.77) | 0.512 | 0.477 ^b^ |
|  | age, smoking and drinking | 1.15(0.75-1.76) | 0.517 | 0.382 ^b^ |
| **rs10773771**  **TC/CC vs. TT** | age, gender, smoking and drinking | 0.86(0.65-1.14) | 0.292 |  |
|  | age, gender and smoking | 0.86(0.65-1.14) | 0.305 | 0.624 ^b^ |
|  | age, gender and drinking | 0.85(0.65-1.13) | 0.274 | 0.495 ^b^ |
|  | age, smoking and drinking | 0.84(0.64-1.11) | 0.232 | 0.327 ^b^ |

^a^ Likelihood ratio test was used to check the difference between -2*log Likelihood of the two models

^b^ Compared to the first model including age, gender, smoking and drinking
